# Pharmacokinetics of first-line tuberculosis drugs rifampin, isoniazid, ethambutol, and pyrazinamide during pregnancy and postpartum with and without efavirenz-based antiretroviral treatment: IMPAACT P1026s study

**DOI:** 10.1128/aac.00052-25

**Published:** 2025-07-31

**Authors:** Marije Van Schalkwyk, Adrie Bekker, Eric Decloedt, Jiajia Wang, Gerhard B. Theron, Mark F. Cotton, Ahizechukwu C. Eke, Tim R. Cressey, Deo Wabwire, David E. Shapiro, Kira Bacon, Kevin Knowles, Kathleen George, Renee Browning, Nahida Chakhtoura, Kittipong Rungruengthanakit, Lubbe Wiesner, Edmund V. Capparelli, Alice M. Stek, Mark Mirochnik, Brookie M. Best, On behalf of IMPAACT P1026s Protocol Team

**Affiliations:** 1 Division of Adult Infectious Diseases, Department of Medicine, Family Center for Research with Ubuntu, Stellenbosch University and Tygerberg Hospital, Cape Town, South Africa; 2 Department of Pediatrics and Child Health, Family Center for Research with Ubuntu, Stellenbosch University and Tygerberg Hospital, Cape Town, South Africa; 3 Division of Clinical Pharmacology, Department of Medicine, Stellenbosch University and Tygerberg Hospital, Cape Town, South Africa; 4 Department of Biostatistics, Harvard T.H. Chan School of Public Health, Center for Biostatistics in AIDS Research 248511 , Boston, Massachusetts, USA; 5 Department of Obstetrics and Gynecology, Stellenbosch University 26697, Cape Town, South Africa; 6 Division of Maternal Fetal Medicine and Clinical Pharmacology, Department of Gynecology and Obstetrics, Johns Hopkins University School of Medicine 1500, Baltimore, Maryland, USA; 7 AMS-PHPT Research Collaboration, Faculty of Associated Medical Sciences, Chiang Mai University 26682, Chiang Mai, Thailand; 8 MU-JHU Research Collaboration, Makerere University 623287, Kampala, Central Region, Uganda; 9 Frontier Science Foundation 2402, Amherst, New York, USA; 10 FHI 360 1311 , Durham, North Carolina, USA; 11 National Institute of Allergy and Infectious Diseases, NIH 35037, Bethesda, Maryland, USA; 12 Maternal and Pediatric Infectious Disease Branch, Eunice Kennedy Shriver National Institute of Child Health and Human Development (NICHD) 35040, Bethesda, Maryland, USA; 13 Research Institute for Health Sciences, Chiang Mai University 551431, Chiang Mai, Thailand; 14 Division of Clinical Pharmacology, Department of Medicine, University of Cape Town 71984, Cape Town, South Africa; 15 Skaggs School of Pharmacy and Pharmaceutical Sciences, University of California 15500, San Diego, California, USA; 16 Department of Pediatrics, School of Medicine, University of California 12219 , San Diego, California, USA; 17 Division of Maternal Fetal Medicine, Department of Obstetrics and Gynecology, University of Southern California School of Medicine, Los Angeles, California, USA; 18 Division of Neonatology, Department of Pediatrics, Boston University Chobanian and Avedisian School of Medicine, Boston, Massachusetts, USA; St George's, University of London, London, United Kingdom

**Keywords:** rifampin, isoniazid, ethambutol, pyrazinamide, efavirenz, antiretroviral therapy, pregnancy, drug-susceptible tuberculosis, pharmacokinetics

## Abstract

The pharmacokinetics (PK) of antituberculosis drugs may be altered by both pregnancy-induced physiological changes and drug interactions in individuals living with HIV who develop tuberculosis. Within the multicenter International Maternal Pediatric Adolescent AIDS Clinical Trials Network P1026s study, we assessed the PK of rifampin, isoniazid, ethambutol, and pyrazinamide during pregnancy and postpartum (PP) in women on efavirenz-based antiretroviral therapy (ART). Results were compared to a previously published non-HIV group and described minimum targets. World Health Organization-recommended daily doses of antituberculosis and ART medications were administered, followed by PK sampling of all antituberculosis drugs over 24 h during the second trimester (2T), third trimester (3T), and 2–8 weeks PP. PK parameters were characterized using noncompartmental analysis, and comparisons were made among stages of pregnancy and between groups using geometric mean ratios with 90% confidence intervals. Twenty-two participants were enrolled, and PK data were available for 12, 20, and 13 participants in 2T, 3T, and PP, respectively. While no significant difference in rifampin exposure between pregnancy and postpartum was detected, the median area-under-the-plasma-concentration-time-curve up to 24 h post-dose (AUC_0–24_) and *C*_max_ were below target during each period and were 42% and 35% lower in 3T than the non-HIV group. No significant difference in isoniazid exposure was found between pregnancy and PP or between the groups. Ethambutol and pyrazinamide AUC_0–24_ and *C*_max_ in 2T and 3T were similar between the groups. In both groups, pyrazinamide *C*_max_ was above target in all periods. The clinical relevance of lower rifampin exposure in pregnant women requiring tuberculosis treatment while on efavirenz should be determined.

## INTRODUCTION

Tuberculosis (TB) and HIV infections remain global emergencies with co-infection frequently occurring (1). In 2023, an estimated 10.7 million people developed TB disease worldwide, 3.6 million of whom were adult women (1) mainly of reproductive age. Pregnancy predisposes to TB acquisition due to temporary immune suppression, with an increased incidence of TB disease observed in the postpartum (PP) period (2, 3). Of concern is an associated threefold increased maternal mortality rate in pregnant women living with HIV (PWLHIV) who develop TB disease during pregnancy and postpartum (4, 5). The reasons for poorer outcomes in PWLHIV with TB disease are manifold.

Pregnancy induces changes in plasma volume, cardiac output, gastric absorption, and renal filtration (6) along with altered expression of hepatic drug-metabolizing enzymes, in particular, progesterone-induced induction of CYP3A peaking specifically in the third trimester (3T) (7). These physiological changes can impact the pharmacokinetics (PK) of TB drugs during pregnancy. Achieving optimal therapeutic concentrations of TB drugs is essential as it predicts favorable treatment outcomes (8). Additionally, concurrent treatment with efavirenz (EFV)-based antiretroviral therapy (ART) can further impact TB drug concentrations, due to well-described drug-drug interactions (DDIs), which may reduce isoniazid (INH) exposure (9).

The World Health Organization (WHO)-TB dosing guidelines do not differentiate between pregnant and non-pregnant adults, recommending rifampin (RIF), INH, ethambutol (EMB), and pyrazinamide (PZA) as first-line antituberculosis therapy for drug-susceptible TB (DS-TB) disease using standard weight-banded dosing (10). Limited PK data are available to guide first-line TB dosing recommendations during pregnancy (11), with even fewer data for PWLHIV who develop TB during pregnancy.

We evaluated the PK of RIF, INH, EMB, and PZA in PWLHIV treated with EFV-based ART during their TB treatment.

## RESULTS

### Participant characteristics

Between November 2014 and March 2019, 22 PWLHIV on EFV-based ART and treated with first-line TB drugs were enrolled and had evaluable PK data. Maternal and infant clinical characteristics are summarized in Table 1 and include data from a previously published non-HIV comparator group (11). Of the 22 PWLHIV on EFV-based ART, 9 (41%) women were slow INH metabolizers, 8 (36%) intermediate, and 5 (23%) fast metabolizers according to their N-acetyltransferase 2 (NAT2) polymorphism profiles. No significant difference in the proportions of each NAT2 classification was found between the groups. Similarly, no significant difference was found in the proportions of participants with solute carrier organic anion transporter family member 1B1 (SLCO1B1) polymorphisms that could affect RIF exposure between the groups.

**TABLE 1:** Maternal and infant clinical characteristics^*f*^

	*n* (%) or Median (range)	*n* (%) or Median (range)	*P*-value
Maternal demographics	With EFV (*n* = 22)	Non-HIV comparator (*n* = 27)	
Age at delivery (years)	31.6 (20.7–38.0)	26.1 (16.2–39.4)	*0.03* ^*a*^
Weight at PK sampling time (kg)			
2T	57.3 (48.0–96.5) (*n* = 12)	61.2 (47.4–89.3) (*n* = 17)	0.77^*a*^
3T	59.4 (49.2–99.4) (*n* = 20)	57.0 (46.1–92.3) (*n* = 21)	0.77^*a*^
PP	54.3 (42.7–68.9) (*n* = 13)	58.9 (48.0–75.4) (*n* = 14)	0.43^*a*^
PK sampling time (gestational age or PP weeks)			
2T	25.8 (21.0–27.0) (*n* = 12)	24.9 (20.0–28.9) (*n* = 17)	0.10^*a*^
3T	32.6 (28.4–37.9) (*n* = 20)	32.9 (30.1–36.9) (*n* = 21)	0.46^*a*^
PP	4.7 (2.3–5.9) (*n* = 13)	4.9 (2.3–7.1) (*n* = 14)	0.39^*a*^
Country			
South Africa	9 (41)	10 (37)	
Thailand	2 (9)	9 (33)	
Uganda	9 (41)	0 (0)	
Tanzania	0 (0)	3 (11)	
Botswana	0 (0)	2 (7)	
Brazil	1 (5)	2 (7)	
USA	1 (5)	1 (4)	
NAT2 metabolizer type			0.69^*b*^
Slow	9 (41)	13 (54)^*c*^	
Intermediate	8 (36)	7 (29)	
Fast	5 (23)	4 (17)	
SLCO1B1 SNPs^*d*^			
rs2306283 (388A>G: AA / AG / GG)	0 (0) / 10 (56) / 8 (44) (*n* = 18)	3 (14) / 14 (67) / 4 (19) (*n* = 21)	0.09^*b*^
rs11045819 (463C>A: GG / GT)	17 (94) / 1 (6) (*n* = 18)	20 (95) / 1 (5) (*n* = 21)	1.00^*b*^
rs4149056 (521T>C: TT / CT)	15 (83) / 3 (17) (*n* = 18)	18 (86) / 3 (14) (*n* = 21)	1.00^*b*^
rs4149032 (38664C>T: CC / CT / TT)	4 (24) / 7 (41) / 6 (35) (*n* = 17)^*e*^	5 (24) / 6 (29) / 10 (48) (*n* = 21)	0.71^*b*^
Antituberculosis drug formulation			
FDC	19 (86)	18 (67)	0.18^*b*^
Individual tablets	3 (14)	9 (33)	
Concomitant antiretroviral regimen			
2T 3TC / FTC / TDF	5 (42) / 7 (58) / 12 (100)		
3T 3TC / FTC / TDF / AZT	12 (60) / 8 (40) / 19 (95) / 1 (5)		
PP 3TC / FTC / TDF / AZT	8 (62) / 5 (38) / 12 (92) / 1 (8)		
CD4 (cells/mm^3^)			
2T	274 (46–695)		
3T	264 (51–999)		
Delivery	290 (35–735)		
PP	268 (42–1,133)		
HIV-1 RNA (copies/mL)			
2T ≤50 / ≤400	5 (42) / 10 (83)		
3T ≤50 / ≤400	14 (70) / 17 (85)		
Delivery ≤50 / ≤400	14 (67) / 17 (77)		
PP ≤50 / ≤400	8 (62) / 9 (69)		
*Infant demographics*			
Gestational age (weeks)	38.9 (26.9–41.7)	38.6 (28.1–41.6)	0.84^*a*^
Birth weight (g)	2,895 (830–4,400) (*n* = 21)	3,100 (1,195–3,960)	0.60^*a*^

^*a*^ Wilcoxon rank-sum test. Values in italic type are significant *P*-values (<0.05).

^*b*^ Fisher’s exact test.

^*c*^ Three participants were excluded from the analysis as not on INH within the required dosing range.

^*d*^ *n* = number of consenting participants analyzed.

^*e*^ One failed amplification; 2T, second trimester; SNP, single nucleotide polymorphism; FDC, fixed-dose combination tablet; 3TC, lamivudine; FTC, emtricitabine; TDF, tenofovir disoproxil fumarate; AZT, zidovudine.

^*f*^ Empty cells indicates not applicable.

**Fig 1:**
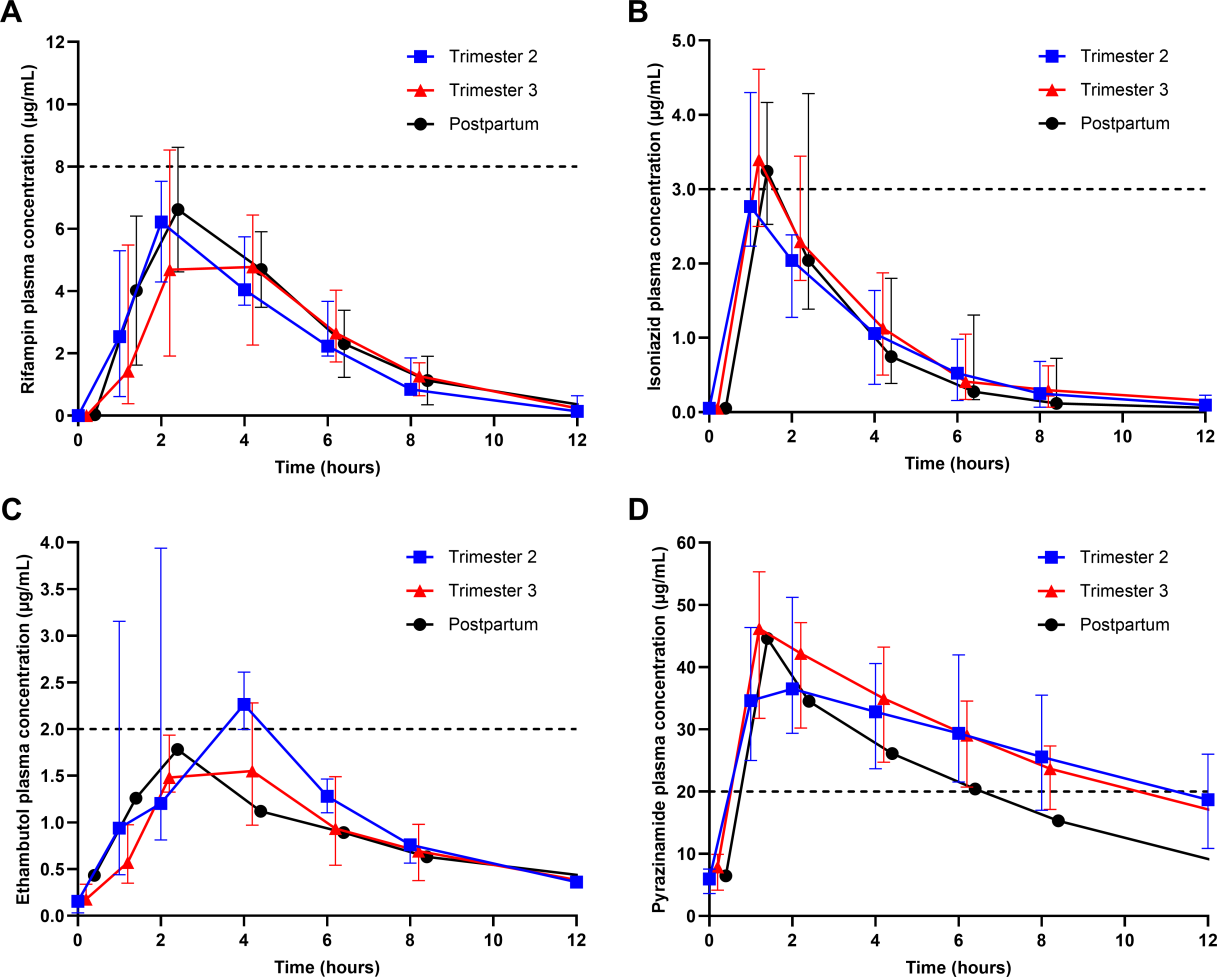
Median plasma concentration-time profiles of (**A**) RIF, (**B**) INH, (**C**) EMB, and (D) PZA during 2T, 3T, and PP (error bars indicate the interquartile range [IQR]). The minimum target *C*_max_ of each drug is represented by the horizontal dashed lines.

**Fig 2:**
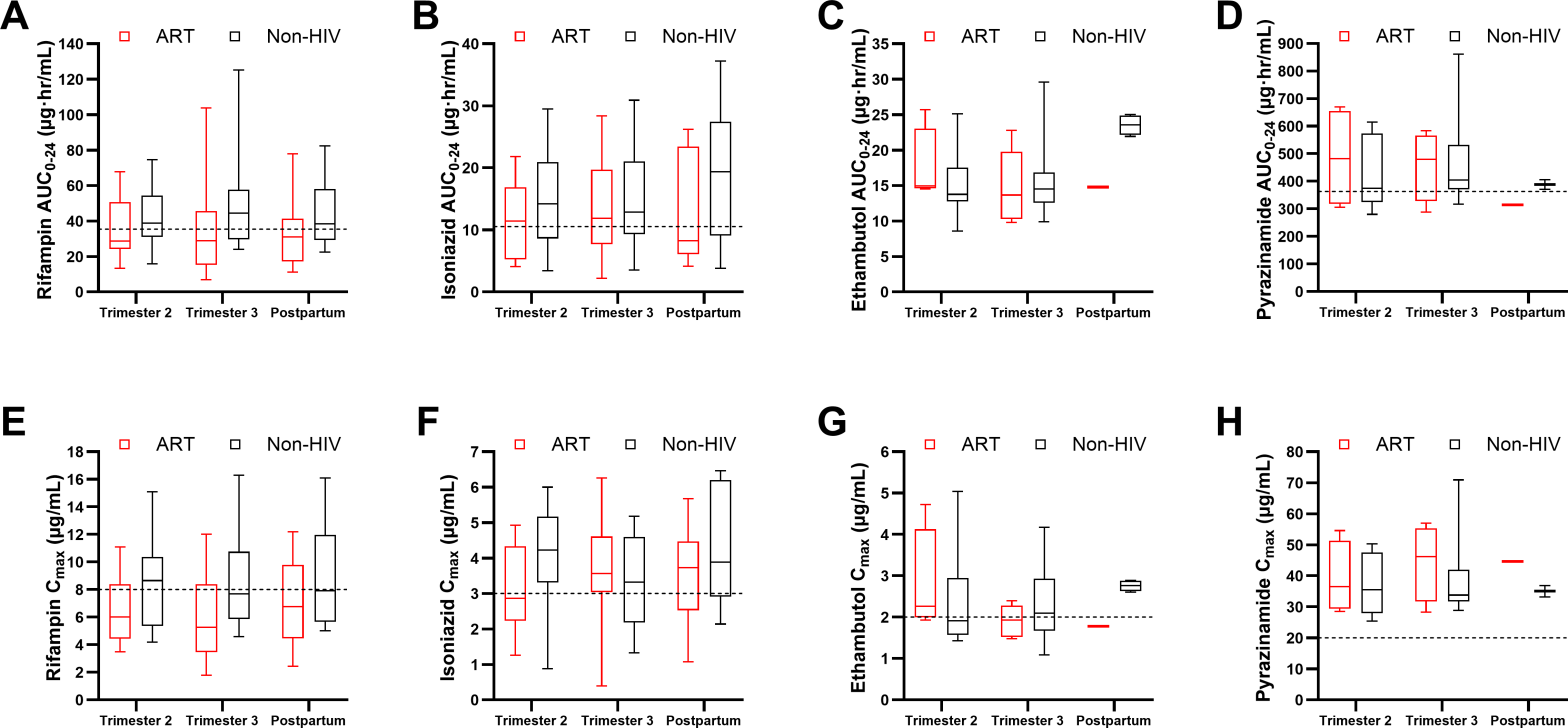
Box and whisker plots comparing plasma AUC_0–24_ of (**A**) RIF, (**B**) INH, (**C**) EMB, and (**D**) PZA and *C*_max_ of (**E**) RIF, (**F**) INH, (**G**) EMB, and (**H**) PZA, respectively, during 2T, 3T, and PP (median, IQR, and range). The minimum targets are represented by the horizontal dashed lines.

Paired pregnancy and PP PK data on RIF and INH were available for 4 of 12 women with second-trimester (2T) visits and 12 of 20 women with 3T visits. Twenty-one women were sampled on more than one occasion (2T, 3T, or PP), and 11 women had both 2T and 3T data. Only one participant was on EMB and PZA in the PP period due to the short 2-month duration of intensive phase TB treatment. The median drug concentrations vs time curves per sampling time point are shown in Fig. 1. PK parameters for RIF and INH are presented in Tables 2 and 3, including within-participant comparisons between the pregnancy trimesters and PP. Corresponding trimester comparisons with the non-HIV group are shown in Table 4. PK parameters for EMB and PZA are presented in Tables 5 and 6, including corresponding trimester comparisons with the non-HIV group. Differences in distributions of area-under-the-plasma-concentration-time-curve up to 24 h post-dose (AUC_0–24_) and *C*_max_ for each drug between each treatment group are shown in Fig. 2.

**TABLE 2:** Maternal RIF dose, PK parameters, and target attainment^*f*^

	Median value for EFV group (IQR)*^a^*			2T / PP comparison^*b*^			3T / PP comparison^*b*^		
	2T *n* = 12	3T *n* = 20	PP *n* = 13	GMR (90% CI)	*n*	*P*-value	GMR (90% CI)	*n*	*P*-value
Dose (mg/kg)	9.76 (8.12–10.48)	9.59 (8.05–10.26)	10.81 (9.64–11.05)		4	0.614^*c*^		12	0.461^*c*^
AUC_0–24_ (µg·h/mL)	28.70 (24.61–49.36)	28.99 (16.16–43.96)	30.91 (17.89–41.25)	1.29 (0.81–2.06)	4		0.94 (0.72–1.23)	12	0.850
CL/F (L/h)	19.05 (12.05–24.36)	18.13 (13.74–32.99)	19.41 (14.46–27.32)	0.78 (0.49–1.24)	4		1.09 (0.85–1.39)	12	0.301
*T*_max_ (h)	2 (1–4)	3 (1–6)	2 (1–4)						
*T*_½_ (h)	1.73 (1.60–2.25)	1.52 (1.44–2.07)	1.58 (1.15–1.92)	0.86 (0.43–1.74)	4		1.08 (0.86–1.36)	12	0.380
*C*_max_ (µg/mL)	6.54 (4.60–8.77)	5.50 (3.39–8.46)	6.75 (4.49–9.67)	1.22 (0.78–1.89)	4		0.87 (0.70–1.08)	12	0.470
*C*_0_ (µg/mL)	BQL	BQL	BQL						
*C*_12_ (µg/mL)	0.14 (BQL–0.57)	0.18 (BQL–0.40)	BQL (BQL–0.32)	2.15 (0.77–6.00)	4		1.32 (0.84–2.07)	8	0.641
*C*_24_ (µg/mL)	BQL	BQL	BQL						
*n* (%) *C*_max_ <8 µg/mL target^*d*^	8 (67%)	14 (70%)	7 (54%)						
*n* (%) AUC_0–24_ <35.4 (µg·h/mL) target^*e*^	7 (58%)	12 (60%)	8 (62%)						

^*a*^ Summary statistics for 2T, 3T and PP presented as median (IQR), except *T*_max_ which is presented as median (range).

^*b*^ Within-participant comparisons between pregnancy and PP presented as geometric mean ratios (GMR) with a 90% confidence interval (CI) and Wilcoxon signed rank test *P*-value when *n* ≥ 5.

^*c*^ Paired *t*-test *P*-value.

^*d*^ Reference (12).

^*e*^ Reference (13); CL/F, apparent oral clearance; *T*_max_, time to maximum plasma concentration; *T*_1/2_, half-life; *C*_max_, maximum plasma concentration; *C*_0_, pre-dose concentration; *C*_12_, 12 h post-dose concentration; *C*_24_, 24 h post-dose concentration; BQL, below quantification limit.

^*f*^ Empty cells indicates not applicable.

**TABLE 3:** Maternal INH dose, PK parameters, and target attainment^*f*^

	Median value for EFV group (IQR)*^a^*			2T / PP comparison^*b*^			3T / PP comparison^*b*^		
	2T *n* = 12	3T *n* = 20	PP *n* = 13	GMR (90% CI)	*n*	*P*-value	GMR (90% CI)	*n*	*P*-value
Dose (mg/kg)	4.88 (4.06–5.24)	4.89 (4.20–5.13)	5.41 (4.82–5.52)		4	0.614^*c*^		12	0.461^*c*^
AUC_0–24_ (µg·h/mL)	11.42 (5.31–16.79)	11.87 (7.69–19.48)	8.28 (6.83–23.36)	0.88 (0.75–1.04)	4		1.01 (0.89–1.15)	12	0.733
CL/F (L/h)	28.13 (17.71–43.05)	22.15 (15.41–38.68)	36.22 (12.84–42.19)	1.14 (0.96–1.34)	4		1.01 (0.90–1.14)	12	0.910
*T*_max_ (h)	1 (1, 2)	1 (1–4)	1 (1, 2)						
*T*_½_ (h)	2.13 (1.58–3.00)	2.60 (1.44–2.87)	1.85 (1.23–2.83)	1.08 (0.79–1.48)	4		0.97 (0.84–1.13)	12	0.850
*C*_max_ (µg/mL)	2.87 (2.24–4.03)	3.56 (3.08–4.60)	3.73 (2.61–4.22)	0.94 (0.76–1.16)	4		1.08 (0.89–1.31)	12	0.791
*C*_0_ (µg/mL)	BQL	BQL	BQL						
*C*_12_ (µg/mL)	0.09 (BQL–0.23)	0.15 (BQL–0.28)	BQL (BQL–0.31)	0.90 (0.70–1.16)	4		0.96 (0.78–1.17)	6	0.156
*C*_24_ (µg/mL)	BQL	BQL	BQL						
*n* (%) *C*_max_ <3 µg/mL target^*d*^	7 (58%)	4 (20%)	6 (46%)						
*n* (%) AUC_0–24_ <10.52 (µg·h/mL) target^*e*^	6 (50%)	9 (45%)	8 (62%)^*f*^						

^*a*^ Summary statistics for 2T, 3T and PP presented as median (IQR), except *T*_max_ which is presented as median (range).

^*b*^ Within-participant comparisons between pregnancy and PP presented as geometric mean ratios (GMR) with a 90% confidence interval (CI) and Wilcoxon signed rank test *P*-value when *n* ≥ 5. Values in italic type are significant *P*-values (<0.1).

^*c*^ Paired *t*-test *P*-value.

^*d*^ Reference (12).

^*e*^ Reference (14); CL/F, apparent oral clearance; *T*_max_, time to maximum plasma concentration; *T*_1/2_, half-life; *C*_max_, maximum plasma concentration; *C*_0_, pre-dose concentration; *C*_12_, 12 h post-dose concentration; *C*_24_, 24 h post-dose concentration; BQL, below quantification limit.

^*f*^ Empty cells indicates not applicable.

**TABLE 4:** Comparison of RIF and INH PK parameters between treatment groups with / without EFV coadministration per trimester^*c*^

	RIF						INH					
	2T comparison with / without EFV^*a*^ (*n* = 12 / *n* = 16)		3T comparison with / without EFV^*a*^ (*n* = 20 / *n* = 20)		PP comparison with / without EFV^*a*^ (*n* = 13 / *n* = 13)		2T comparison with / without EFV^*a*^ (*n* = 12 / *n* = 14)		3T comparison with / without EFV^*a*^ (*n* = 20 / *n* = 19)		PP comparison with / without EFV^*a*^ (*n* = 13 / *n* = 12)	
	GMR (90% CI)	*P*-value	GMR (90% CI)	*P*-value	GMR (90% CI)	*P*-value	GMR (90% CI)	*P*-value	GMR (90% CI)	*P*-value	GMR (90% CI)	*P*-value
Dose (mg/kg)		0.857^*b*^		0.601^*b*^		*0.013* ^*b*^		0.619^*b*^		0.881^*b*^		0.192^*b*^
AUC_0–24_ (µg·h/mL)	0.81 (0.61–1.08)	0.226	0.58 (0.42–0.81)	*0.008*	0.73 (0.53–1.00)	0.104	0.75 (0.49–1.13)	0.236	0.86 (0.61–1.21)	0.458	0.67 (0.42–1.07)	0.151
CL/F (L/h)	1.24 (0.94–1.63)	0.192	1.77 (1.32–2.38)	*0.002*	1.48 (1.09–2.02)	*0.039*	1.28 (0.84–1.95)	0.329	1.17 (0.84–1.64)	0.436	1.51 (0.95–2.41)	0.140
*T*_½_ (h)	1.10 (0.82–1.48)	0.595	0.76 (0.64–0.91)	*0.012*	0.94 (0.73–1.21)	0.685	1.08 (0.83–1.40)	0.626	0.87 (0.69–1.10)	0.325	0.84 (0.59–1.21)	0.430
*C*_max_ (µg/mL)	0.81 (0.63–1.06)	0.187	0.65 (0.50–0.85)	*0.009*	0.80 (0.59–1.08)	0.222	0.78 (0.57–1.07)	0.193	1.03 (0.78–1.34)	0.876	0.79 (0.59–1.06)	0.188
*C*_0_ (µg/mL)	0.96 (0.74–1.25)	0.794	0.95 (0.68–1.34)	0.815	1.11 (0.86–1.44)	0.491	0.84 (0.60–1.16)	0.367	1.11 (0.78–1.58)	0.625	0.89 (0.64–1.24)	0.561
*C*_12_ (µg/mL)	0.72 (0.34–1.50)	0.449	0.41 (0.23–0.73)	*0.013*	0.63 (0.29–1.35)	0.309	0.96 (0.52–1.77)	0.905	0.80 (0.48–1.35)	0.481	0.56 (0.25–1.26)	0.233

^*a*^ Comparisons of corresponding trimesters between treatment groups, presented as univariate mixed effect model geometric mean ratios (GMR) with a 90% confidence interval (CI) and *P*-value. Values in italic type are significant *P*-values (<0.1).

^*b*^ Two-sample *t*-test *P*-value.; CL/F, apparent oral clearance; *T*_½_, half-life; *C*_max_, maximum plasma concentration; *C*_0_, pre-dose concentration; *C*_12_, 12 h post-dose concentration.

^*c*^ Empty cells indicates not applicable.

**TABLE 5:** Maternal EMB dose, PK parameters, target attainment, and comparison between treatment groups with / without EFV coadministration per trimester^*e*^

	Median value for EFV group (IQR)*^a^*			2T comparison with / without EFV^*b*^ (*n* = 4 / *n* = 8)		3T comparison with / without EFV^*b*^ (*n* = 5 / *n* = 11)	
	2T *n* = 4	3T *n* = 5	PP *n* = 1	GMR (90% CI)	*P*-value	GMR (90% CI)	*P*-value
Dose (mg/kg)	17.58 (15.11–19.87)	17.03 (16.77-20.37)	17.67		0.814^*c*^		0.307^*c*^
AUC_0–24_ (µg·h/mL)	14.94 (14.68–20.35)	13.67 (10.75–6.71)	14.77	1.17 (0.84–1.63)	0.412	0.93 (0.69–1.25)	0.658
CL/F (L/h)	64.47 (48.86–74.92)	76.76 (49.38–80.47)	55.87	0.85 (0.64–1.14)	0.340	1.03 (0.76–1.41)	0.869
*T*_max_ (h)	4 (2–4)	4 (2–4)	2				
*T*_½_ (h)	5.85 (5.33–6.94)	8.25 (7.27–8.44)	9.04	1.51 (1.22–1.88)	*0.006*	2.05 (1.69–2.50)	*<0.001*
*C*_max_ (µg/mL)	2.27 (2.06–3.53)	1.93 (1.55–2.17)	1.78	1.22 (0.77–1.95)	0.453	0.89 (0.64–1.23)	0.529
*C*_0_ (µg/mL)	0.16 (0.08–0.19)	0.18 (0.18–0.28)	0.43	0.81 (0.41–1.58)	0.577	1.36 (0.93–1.99)	0.178
*C*_12_ (µg/mL)	0.36 (0.32–0.37)	0.37 (0.29–0.43)	0.42	0.89 (0.64–1.24)	0.529	0.95 (0.65–1.39)	0.820
*C*_24_ (µg/mL)	0.15 (0.11–0.20)	0.20 (0.18–0.21)	0.23				
*n* (%) *C*_max_ <2 µg/mL target^*d*^	1 (25%)	3 (60%)	1 (100%)				

^*a*^ Summary statistics for 2T/3T and PP presented as median (IQR), except *T*_max_ which is presented as median (range).

^*b*^ Comparisons of corresponding trimesters between treatment groups, presented as univariate mixed effect model geometric mean ratios (GMR) with a 90% confidence interval (CI) and *P*-value. Values in italic type are significant *P*-values (<0.1).

^*c*^ Two-sample *t*-test *P*-value.

^*d*^ Reference (12); CL/F, apparent oral clearance; *T*_max_, time to maximum plasma concentration; *T*_½_, half-life; *C*_max_, maximum plasma concentration; *C*_0_, pre-dose concentration; *C*_12_, 12 h post-dose concentration; *C*_24_, 24 h post-dose concentration.

^*e*^ Empty cells indicates not applicable.

**TABLE 6:** Maternal PZA dose, PK parameters, and target attainment, and comparison between treatment groups with / without EFV coadministration per trimester^*f*^

	Median value for EFV group (IQR)*^a^*			2T comparison with / without EFV^*b*^ (*n* = 4 / *n* = 6)		3T comparison with / without EFV^*b*^ (*n* = 4 / *n* = 9)	
	2T *n* = 4	3T *n* = 4	PP *n* = 1	GMR (90% CI)	*P*-value	GMR (90% CI)	*P*-value
Dose (mg/kg)	25.57 (21.98–28.91)	27.20 (24.58–30.41)	25.70		0.745^*c*^		0.105^*c*^
AUC_0–24_ (µg·h/mL)	482.88 (331.97–638.58)	480.15 (367.88–548.46)	314.64	1.13 (0.75–1.69)	0.598	1.00 (0.71–1.39)	0.985
CL/F (L/h)	2.99 (2.51–4.29)	3.35 (2.93–3.87)	3.81	0.92 (0.69–1.23)	0.613	1.03 (0.83–1.28)	0.801
*T*_max_ (h)	2 (2)	1 (1)	1				
*T*_½_ (h)	7.13 (6.21–9.15)	6.79 (6.42–7.57)	4.74	0.90 (0.69–1.18)	0.491	0.77 (0.58–1.01)	0.116
*C*_max_ (µg/mL)	36.50 (30.20–47.85)	46.20 (35.25–53.60)	44.60	1.05 (0.76–1.46)	0.789	1.14 (0.85–1.55)	0.442
*C*_0_ (µg/mL)	5.96 (3.99–7.40)	7.88 (5.02–9.60)	6.46	1.13 (0.66–1.93)	0.684	1.32 (0.79–2.20)	0.347
*C*_12_ (µg/mL)	18.70 (11.65–25.40)	16.80 (12.62–19.70)	8.47	1.15 (0.71–1.87)	0.601	0.94 (0.65–1.35)	0.757
*C*_24_ (µg/mL)	5.38 (2.87–9.05)	4.89 (3.24–6.15)	1.40				
*n* (%) *C*_max_ <20 µg/mL target^*d*^	0 (0%)	0 (0%)	0 (0%)				
*n* (%) AUC_0–24_ <363 (µg·h/mL) target^*e*^	2 (50%)	1 (25%)	1 (100%)				

^*a*^ Summary statistics for 2T, 3T and PP presented as median (IQR), except *T*_max_ which is presented as median (range).

^*b*^ Comparisons between pregnancy and PP presented as univariate mixed effect model geometric mean ratios (GMR) with a 90% confidence interval (CI) and *P*-value.

^*c*^ Two-sample *t*-test *P*-value.

^*d*^ Reference (12).

^*e*^ Reference (8); CL/F, apparent oral clearance; *T*_max_, time to maximum plasma concentration; *T*_½_, half-life; *C*_max_, maximum plasma concentration; *C*_0_, pre-dose concentration; *C*_12_, 12 h post-dose concentration; *C*_24_, 24 h post-dose concentration.

^*f*^ Empty cells indicates not applicable.

### Rifampin pharmacokinetics

Overall, 12, 20, and 13 women completed 2T, 3T, and PP RIF PK sampling, respectively (Table 2). When comparing 2T and 3T to PP, no significant difference in AUC_0–24_, *C*_max_, or dose-adjusted C_max_ between pregnancy and postpartum was detected, as the 90% confidence interval (CI) for the geometric mean ratios (GMR) included one for both antepartum trimesters in the within-participant comparison to PP. However, the median RIF AUC_0–24_ and median RIF *C*_max_ were below the efficacy target in all sampling periods. During 3T, 70% of all participants did not reach target *C*_max_, and 60% did not reach target AUC_0–24_. No statistical difference in RIF dose was detected between the sampling periods, and the RIF dose was notably higher in the group with *C*_max_ above the minimum cutoff compared with the group with *C*_max_ below the minimum cutoff in 3T (median 10.56 vs 8.87 mg/kg, *P* = 0.006) and PP (median 11.02 vs 9.64 mg/kg, *P* = 0.038) and showed a similar trend in 2T, demonstrating direct correlation. Compared to the previously published non-HIV group (11), the 3T RIF dose did not differ significantly, but the 3T RIF AUC_0–24_ was 42% lower (GMR 0.58 [90% CI 0.42–0.81]; *P =* 0.008), and *C*_max_ was 35% lower (GMR 0.65 [90% CI 0.50–0.85]; *P =* 0.009) with lower trends in 2T and PP, as shown in Table 4. The PP RIF dose was higher in the ART group (median 10.81 vs 9.30 mg/kg, *P* = 0.013), but no significant difference in median RIF AUC_0–24_ and RIF *C*_max_ was detected between the groups. The non-HIV group showed a trend toward more (*n* = 3) participants with SLCO1B1 c.388A>G (*1b) single nucleotide polymorphism (SNP) homozygous variant (AA genotype), as the ART group had none (*P* = 0.09). In the non-HIV group, this AA genotype was associated with higher AUC_0–24_ and *C*_max_ compared to the wild-type GG (Supplemental Material S1 https://doi.org/10.25413/sun.29182202) but had a very small sample size.

### Isoniazid pharmacokinetics

**Fig 3:**
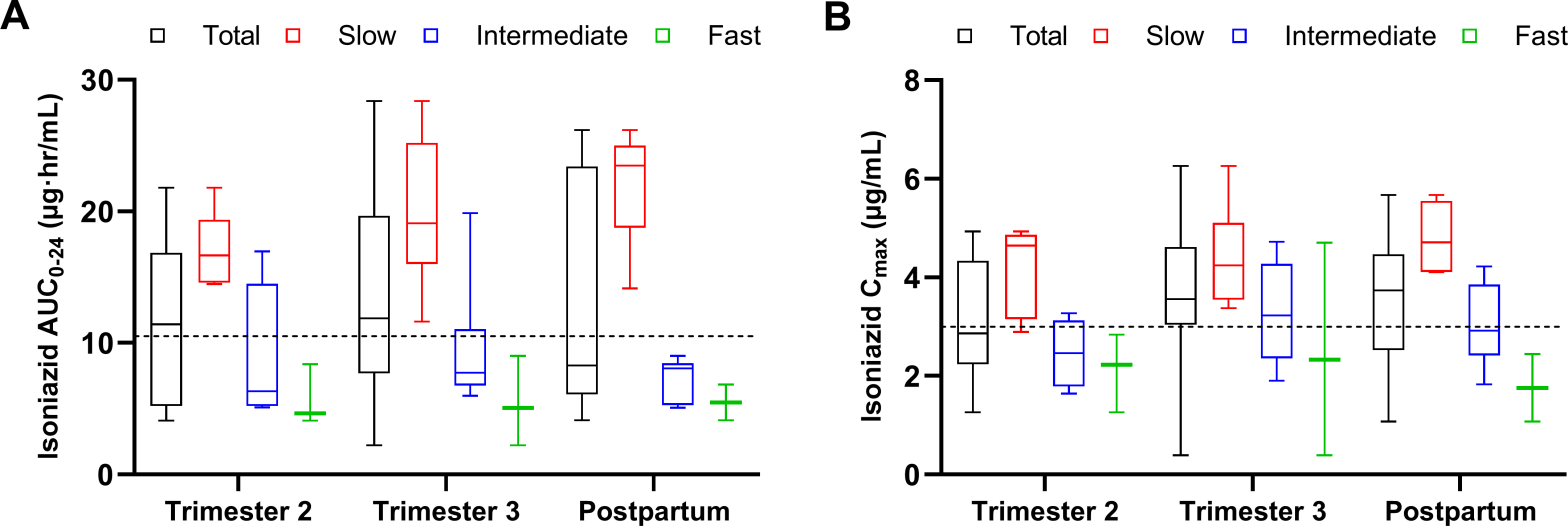
Box and whisker plots showing plasma INH (**A**) AUC_0–24_ and (**B**) *C*_max_ for the total group and per metabolizer type during the 2T and 3T and PP (median, IQR, and range). The minimum target AUC_0–24_ and *C*_max_ are represented by the dashed lines.

A total of 12, 20, and 13 women completed 2T, 3T, and PP INH PK sampling, respectively (Table 3). Compared with PP, the pregnancy INH AUC_0–24_, *C*_max_, and dose-adjusted C_max_ did not differ significantly in both 2T and 3T. Both median INH AUC_0–24_ and *C*_max_ were above target in 3T, but in 2T, the median *C*_max_ was below target, and in PP, the median AUC_0–24_ was below target. In 2T, 58% and 50% of participants did not reach target *C*_max_ and AUC_0–24_, respectively, improved in 3T, but again deteriorated in PP. No difference in INH exposure was found between the groups with and without EFV (Table 4). INH AUC_0–24_ depended on the NAT2 metabolizer type (Fig. 3) with significant differences between the metabolizer groups in 3T and PP (*P-*values 0.055, 0.003, and 0.01 for 2T, 3T, and PP, respectively). INH *C*_max_ was similarly dependent on the metabolizer type (Fig. 3, *P*-values 0.013, 0.048, and 0.002 for 2T, 3T, and PP, respectively). Slow metabolizers had the highest median AUC_0–24_ and *C*_max_ at all timepoints. None of the five fast metabolizers reached the target AUC_0–24_ in any period, with only one reaching the *C*_max_ target concentration in 3T.

### Ethambutol pharmacokinetics

Four, five, and one women completed 2T, 3T, and PP EMB PK sampling, respectively (see Table 5). While median *C*_max_ was marginally above and below target in 2T and 3T, 25% and 60% of participants did not reach target *C*_max_, respectively. Within-participant and between-trimester comparisons could not be performed. Compared to the historical non-HIV group, the EMB AUC_0–24_ and *C*_max_ in both trimesters did not differ significantly, but 2T *T*_½_ was 51% higher (GMR 1.51 [90% CI 1.22–1.88]; *P =* 0.006) and 3T *T*_½_ was 105% higher (GMR 2.05 [90% CI 1.69–2.50]; *P <*0.001), while the dosing range was similar.

### Pyrazinamide pharmacokinetics

Four, four, and one women completed 2T, 3T, and PP PZA PK sampling, respectively (see Table 6). All participants had a PZA *C*_max_ above the minimum target in all the sampling timepoints, but 50% did not reach target AUC_0–24_ in 2T, nor did 25% in 3T and the single postpartum participant. Within-participant and between-trimester comparisons could not be performed. No significant differences were found in PZA PK parameters between the ART group and the historical non-HIV group.

### Delivery samples

One pair of maternal and cord blood samples collected at delivery had detectable levels for all drugs, and cord/maternal concentration ratios were 0.42 for RIF, 1.07 for INH, 0.69 for EMB, and 0.96 for PZA. No statistical analysis was performed.

### Maternal and infant safety outcomes

Maternal and infant safety events in the ART group are summarized in Table 7. Eleven (50%) women experienced one or more grade 3 or greater adverse events (AEs) after study entry, including five (23%) cases of grade 3 anemia (Hb 6.5 to <8.5 g/dL) at study entry and two (9%) postpartum. Eight (36%) participants had grade 2 anemia (Hb 8.5 to <9.5 g/dL). No drug-induced liver injury (DILI) was reported, but one (5%) grade 2 TB drug-related skin eruption had occurred before study entry. After short-term TB drug interruption, the lesions resolved, with all four TB drugs reintroduced successfully in a staggered fashion at standard dosing ranges. Adverse pregnancy outcomes occurred in five (23%) participants, including three (14%) preterm deliveries, of which one was severe (<32 weeks gestation) due to placental abruption at 27 weeks gestation. All preterm deliveries were classified as unrelated to treatment. Other adverse pregnancy outcomes included one intrauterine fetal death due to maternal malaria and one obstructed labor. Maternal TB treatment outcomes were not collected.

**TABLE 7:** Maternal and infant safety outcomes and TB treatment-related AEs^*c*^

Maternal events^*a*^ (22 mothers in total)	*n* (%)	Infant events^*a*^ (21 infants in total)	*n* (%)
Mothers with one or more ≥ grade 3 AE	11 (50)	Infants with one or more ≥ grade 3 AE	6 (29)
Malaria in pregnancy	1 (5)	Neonatal death (intracranial hemorrhage and RDS)	1 (5)
Urinary tract infection	1 (5)	Meconium aspiration syndrome	1 (5)
Gastritis	2 (9)	Neonatal anemia	1 (5)
Anemia	7 (32)	Neonatal hypoglycemia	2 (10)
		Hyperthermia	1 (5)
TB treatment-related events		Gastroenteritis (with neutropenia)	1 (5)
Drug-induced skin rash^*b*^	1 (5)		
Adverse pregnancy outcomes		Adverse birth weight outcomes	
Intrauterine fetal death (malaria)	1 (5)	Low birth weight (<2,500 g)	4 (19)
Preterm delivery	3 (14)	Very low birth weight (<1,500–1,000 g)	0 (0)
Moderate to late preterm (32 to <37 weeks)	2 (9)	Extremely low birth weight (<1,000 g)	1 (5)
Very preterm (28 to <32 weeks)	0 (0)	SGA	4 (19)
Extremely preterm (<28 weeks)	1 (5)	IUGR	1 (5)
Abruptio placenta	1 (5)		
Obstructed labor	1 (5)		

^*a*^ Associated events/symptoms are counted as one diagnosis.

^*b*^ Grade 2 and resolved with TB drug desensitization before study entry. RDS, respiratory distress syndrome; SGA, small for gestational age; IUGR, intrauterine growth restriction.

^*c*^ Empty cells indicates not applicable.

Six (29%) infants experienced adverse events grade ≥3 of which none were classified as DS-TB treatment related. One premature infant, born at 27 weeks gestation after abruptio placenta, died due to intraventricular hemorrhage and respiratory distress syndrome. No congenital abnormalities or congenital TB were reported. None of the adverse birth weight outcomes were classified as treatment related.

## DISCUSSION

This study provides critical insights into the PK of WHO-recommended weight-banded doses of first-line TB drugs RIF, INH, EMB, and PZA in PWLHIV on EFV-based ART. We found that the AUC_0–24_ and *C*_max_ of RIF and INH did not differ significantly during pregnancy compared with post-partum in both the ART group and the previously published non-HIV comparator group (11), but in contrast to the non-HIV group, the median RIF AUC_0–24_ and *C*_max_ in the ART group were below therapeutic targets in all sampling timepoints. For participants on EFV-based ART, RIF AUC_0–24_ and *C*_max_ were 42% and 35% lower, respectively, in 3T compared to the non-HIV group. The median INH AUC_0–24_ remained above target during pregnancy, and no significant differences were found in INH exposure between both groups. NAT2 metabolizer genotype determined the INH exposure.

We found no significant difference in RIF exposure between pregnant and non-pregnant states within either group, consistent with prior findings from the Tshepiso study, a population pharmacokinetic (PopPK) model that evaluated RIF PK in a similar cohort of PWLHIV predominantly on EFV-based ART (15). The Tshepiso PopPK model, which sampled 33 participants up to 8 h post-dose at 37 weeks of gestation or at delivery and again at 6 weeks postpartum, predicted only a mild 14% reduction in apparent clearance during pregnancy, with slightly increased exposure in pregnancy not requiring a dose adjustment. However, our ART group had markedly lower median [IQR] 3T AUC_0–24_ (28.99 [16.16–43.96] µg·h/mL) and *C*_max_ (5.50 [3.39–8.46] µg/mL) compared to these model’s estimates (40.8 [27.1–54.2] µg·h/mL and 8.4 [7.1–10.0] µg/mL, respectively). These consistently below-target RIF exposures across pregnancy and postpartum sampling periods suggest that pregnancy-related changes alone do not fully account for these lower levels. Our analysis also showed that 70% of participants in 3T did not reach target C_max_, and 60% did not achieve target AUC_0–24_, reflecting subtherapeutic RIF levels. Comparisons with the non-HIV group revealed that PWLHIV on EFV-based ART had significantly lower RIF AUC_0–24_ (42% lower) and C_max_ (35% lower) in 3T, likely influenced by ART-related DDIs. Although no pharmacogenetic differences in SLCO1B1 polymorphisms (specifically the c.388A>G genotype associated with higher RIF exposure) were observed between groups, the absence of this genotype in our ART group may partly explain the lower RIF exposure. Both HIV infection (16, 17) and EFV-based ART (18) have been reported to lower RIF exposure in non-pregnant populations. However, the interaction has not been consistently described, as other studies could not demonstrate an effect of EFV on RIF exposure (9, 19, 20). RIF undergoes clearance autoinduction via the pregnane X receptor (21), resulting in lower steady-state concentration, while EFV, a substrate of cytochrome P-450 (mainly CYP2B6) and a known inducer of CYP3A4 and P-glycoprotein (22), may further affect RIF metabolism (18). Our data suggest that the combination of late pregnancy physiological changes and EFV exposure could contribute to increased RIF clearance in 3T and postpartum, potentially altering RIF metabolism even after a steady state is reached for both drugs. This underscores the need for dose optimization and suggests that therapeutic drug monitoring (TDM) in PWLHIV on EFV-based ART may be needed to ensure sufficient RIF exposure, particularly in late pregnancy, when drug concentrations are most affected. However, TDM implementation in low-resource TB endemic settings can be challenging.

Our findings of high proportions of participants on EFV-based ART not achieving the minimum *C*_max_ target also align with observations from the Tshepiso cohort (15). Low plasma exposure to rifamycins has been associated with TB treatment failure and the emergence of rifamycin resistance (23), making the significant number of participants with subtherapeutic RIF AUC_0–24_ and *C*_max_ while on EFV particularly concerning. Although it is reassuring that pregnancy itself does not seem to further lower RIF exposure, these results further underscore the potential need for TDM of RIF in pregnancy if on EFV-based ART. Recent studies suggest that higher RIF dosing to reach minimum concentration targets (24, 25) could be achieved by adding an extra RIF tablet to the standard fixed-dose combination (FDC) tablet regimen. Evaluating the exposure, safety, and efficacy of high-dose RIF TB treatment in pregnancy is therefore urgently needed. Although dolutegravir-based ART regimens have become more widely available and preferred as first-line ART regimen, and EFV use has subsequently declined, our findings are still relevant as pharmacy stockouts might necessitate a temporary switch to the EFV-based regimen in low-resource settings. Additionally, there may still be a preference for the single once-daily EFV-based ART FDC in situations where it aligns with the once-daily TB treatment schedule, as dolutegravir requires twice-daily dosing when coadministered with RIF. Increased pill burden in the setting of dual disease is an important obstacle, especially when pregnancy-related nausea and vomiting might involuntarily jeopardize adherence, which underlines the relevance of evaluating the impact of EFV-based ART on TB treatment outcomes in TB-affected pregnancies, despite it no longer being recommended as first-line ART even in high TB-burdened countries. It is currently unclear whether the gain of single-dose ART offsets the potential risk of subtherapeutic RIF exposure.

INH exposure in the ART group did not differ significantly in pregnancy and postpartum, contrasting with our previous observations in a non-HIV group, where INH exposure was 18%–25% lower during pregnancy compared to postpartum (11). This expected reduction in exposure during pregnancy, due to increased glomerular filtration rate (26), and predominant renal excretion of INH , has also been reported in some but not all previous studies. For instance, Gausi et al. observed a 26% increase in INH clearance during pregnancy in a TB prevention cohort of PWLHIV predominantly on EFV-based ART (27). However, other studies have reported no effect of pregnancy on INH exposure. In one study of 29 PWLHIV treated for TB disease, predominantly on EFV-based ART, no difference in INH exposure was seen between pregnancy and postpartum, though NAT2 genotypes were not differentiated (28). Given that NAT2 genetic variations influence INH clearance due to differing acetylation rates (14), within-participant comparisons that account for these pharmacogenomic variations are crucial to understanding pregnancy effects on INH PK. Amaeze et al. developed a PopPK model to examine how the PK of INH changes during pregnancy across different NAT2 phenotypes (29). Their findings indicate that the increased clearance of INH observed during pregnancy is primarily due to increased renal clearance rather than alterations in NAT2 activity, which appeared unchanged during pregnancy. This increase in INH clearance is more pronounced in NAT2 slow metabolizers, who have higher drug exposure. However, we found no significant difference in proportions of slow, intermediate, or fast metabolizers between the treatment groups that could explain the difference in clearance.

We observed no significant differences in INH exposure between the treatment groups across any sampling period, which was unexpected. Bhatt et al. conducted within-participant comparisons of RIF and INH PK before and after initiation of EFV in a non-pregnant cohort (58% male) and reported a 29% reduction in INH exposure after 4 weeks of coadministered EFV (9). In contrast, the median INH AUC_0–24_ and *C*_max_ in our ART group were lower than the reported 14.06 (µg·h/mL) and 4.91 mg/L, respectively, reported by Bhatt et al. Given EFV’s known impact on the acetylation pathway, Chirehwa et al. similarly predicted a 29% reduction in INH exposure, specifically in fast acetylators on EFV-based ART but not in slow acetylators (30). This observation may explain our findings, as both our treatment groups consisted primarily of slow acetylators. Consistent with these studies, none of the fast acetylators in the ART group achieved the AUC_0–24_ target of 10.52 µg·h/mL, and only one participant achieved the minimum target INH *C*_max_ of 3 mg/L. Although the median *C*_max_ in the ART group approached the target in the 2T and exceeded the target in the 3T and PP, the high proportion of participants who did not achieve their target *C*_max_ is concerning, as lower INH levels are associated with poorer clinical and treatment outcomes (12). Additionally, fast acetylators are at a higher risk of underexposure and resistance development (31). A larger sample size, allowing for within-participant analyses between second trimester and postpartum states, is necessary to better ascertain pregnancy’s effects on INH metabolism and to support genotype-informed dosing recommendations in pregnancy.

No established target values for EMB AUC_0–24_ have been described in the literature. In our study, the EMB AUC_0–24_ and *C*_max_ in the ART group during pregnancy were comparable to those in the non-HIV group. The non-HIV group demonstrated higher EMB clearance during pregnancy, resulting in lower EMB AUC_0–24_ and *C*_max_ compared to postpartum, likely due to pregnancy-induced hyperfiltration, as EMB is predominantly eliminated unchanged in the urine. Our findings on EMB AUC_0–24_ and *C*_max_ during pregnancy align with results from Abdelwahab et al., who reported comparable median *C*_max_ values of 1.82 and 2.1 µg/mL, and AUC_0–24_ values of 16.5 and 19.0 µg·h/mL for antepartum and postpartum periods, respectively (28). The EMB PopPK model from this study did not detect any significant effects of pregnancy or EFV coadministration effects on EMB clearance or bioavailability, although it is limited by a similarly low postpartum sample size. Other studies on non-pregnant cohorts, each with variable proportions of female sex and HIV comorbidity, have reported similar (32, 33) or higher (25, 34) values. A larger sample of pregnant and postpartum persons on EMB is urgently needed to allow for a sufficiently powered within-participant analysis to confirm the potential pregnancy effects on EMB PK.

PZA PK parameters in pregnancy for the ART group were not significantly different from those in the non-HIV group. Due to the limited postpartum sample size, we could not fully assess the effects of pregnancy on PZA PK. However, the observed PZA AUC_0–24_ and *C*_max_ were comparable to those reported by Abdelwahab et al., who found median *C*_max_ values of 35.9 and 34.5 µg/mL, and AUC_0–24_ values of 419 and 407 µg·h/mL for antepartum and postpartum participants, respectively (28). The PZA PopPK model in this study did not detect significant effects of pregnancy or EFV coadministration on PZA clearance and bioavailability, though it was similarly constrained by a small postpartum sample size. Other studies in non-pregnant cohorts have reported comparable results (25, 33, 35). Given that PZA undergoes metabolism and elimination through multiple pathways, it is unlikely that pregnancy substantially impacts its exposure. It is reassuring that all participants reached target *C*_max_, and the majority reached target AUC_0–24_, as PZA exposure is the most important predictor of both sputum conversion and sterilizing activity when used in combination with TB therapy (8, 36).

Most AEs reported in the ART group during the study were considered unrelated to TB treatment. While both treatment groups had small sample sizes and included participants of many ethnicities and a spectrum of TB disease severity and treatment duration, it was surprising that not a single case of DILI occurred in the ART group, as the non-HIV group had five (19%) cases (11). Besides TB drug exposure, risk factors for DILI are pregnancy (37), advanced age, female sex, slow acetylator status, malnutrition, and pre-existent liver disease (38). HIV infection has been associated with both higher and lower risk of hepatotoxicity (39, 40). While the ART group was older and had concurrent HIV and additional exposure to EFV, a potentially hepatotoxic drug, this group had less RIF exposure and fewer participants on PZA. These factors might partly explain the lower DILI occurrence. NAT2 metabolizer groups and INH exposure were similar between the groups and are therefore less likely to be contributing factors. Additional pharmacogenetic differences not investigated in our study might also have occurred between the groups, as polymorphisms in several drug-metabolizing enzyme pathways are linked with susceptibility to DILI, such as cytochrome P450 2E1, glutathione S-transferase, and uridine 5'-diphospho-glucuronosyltransferase (41).

Half of the participants experienced one or more adverse events, of which severe anemia at study entry was most common in seven (32%) participants. A recent systematic review found that anemia is detected in 62% of newly diagnosed TB patients (42) and is the most common comorbidity in active TB disease. TB-associated anemia has a multifactorial etiology, including inflammation (anemia of chronic disease) and nutritional deficiency (iron deficiency), of which the latter is also frequent in pregnancy because of increased demand (43).

As both TB disease and anemia in pregnancy are associated with preterm delivery and low birth weight (4, 44–46), high rates were expected, although the rates were lower than in the non-HIV group (11). No congenital abnormalities or TB treatment-related events in infants were reported in the ART group. This is reassuring and supports the fetal safety of DS-TB drugs in pregnancy as summarized by Shiu et al. (47).

Our study has several strengths. First, this is the first intensive PK study sampling in both the second and third trimesters of pregnancy and postpartum where FDC tablets were used for both TB treatment and ART where available according to WHO dosing guidelines, ensuring generalizability of our findings. Second, within-participant comparison using the PP period as control, adjusting for possible pharmacogenetic and other participant-specific influences, was achieved for RIF and INH in more than 10 participants per treatment group, adding strength to the reliability of the univariate mixed-effect model-derived GMRs for these drugs. Third, pharmacogenomic sampling was included in consenting participants, allowing evaluation of possible pharmacogenetic traits that could influence PK findings. Fifth, the long duration of follow-up until 16–24 weeks postpartum enabled us to record AEs related to DS-TB disease as well as DS-TB treatment exposure at regular intervals in both mother and infant.

This study had several limitations. First, the early PP sampling window of 2–8 weeks after delivery might not have accurately captured the postpartum period as we tried to include as many postpartum women as possible before completion of TB treatment, although the median sampling time wasmore than 4.5 weeks from delivery in both groups. Second, despite the early PP sampling window, only one participant of the ART group was on EMB and PZA in the postpartum period due to the short 2-month duration of the TB treatment initiation phase, preventing within-participant comparisons for these drugs. Third, maternal TB treatment outcomes were not collected, nor were minimum inhibitory concentration data available for our participants, which limited the interpretation of the clinical consequences of our PK findings. Fourth, by following WHO weight-banded dosing guidelines with FDCs, some within-participant dosing variation could not be prevented. Concurrently, the TB treatment continuation phase limits RIF dosing to a maximum of 600 mg daily and INH to 300 mg daily, resulting in relative underdosing of participants with weights over 70 kg, although this did not occur frequently. However, the reported apparent oral clearance (CL/F) is dose-corrected, and we observed the same trend as for AUC, strengthening the findings. Fifth, we did not measure free RIF concentrations. RIF is moderately (80%) protein bound, and pregnancy-induced differences in plasma volume and circulating proteins could affect the free fraction, subsequently resulting in clearance differences. Sixth, as TB treatment FDC formulations and meal recommendations were not standardized, heterogeneity in absorption related to formulation, dietary intake at dosing time, and pregnancy-mediated decreased gastric motility may have influenced PK profiles. Lastly, as participants receiving adjusted TB treatment regimens due to treatment-related AEs would not have been eligible for inclusion in this study, selection bias might have occurred in favor of participants tolerating the standard TB regimen for at least 2 weeks before entry, resulting in potential underreporting of treatment-related AEs.

This study was intended to assess whether PWLHIV who received antituberculosis therapy while on EFV-based ART achieved plasma concentrations that met the therapeutic targets and were comparable to those in non-pregnant adults. It is important to emphasize that this PK study was not powered for efficacy or safety outcomes and recruited a heterogeneous population of PWLHIV stable on TB treatment for >2 weeks, with a diverse spectrum of DS-TB and HIV disease at entry.

In conclusion, we found that in PWLHIV receiving EFV-based ART during treatment for DS-TB disease, RIF and INH AUC_0–24_ and *C*_max_ in pregnancy did not differ significantly compared with postpartum, and no pregnancy effect was identified. However, median RIF AUC_0–24_ and *C*_max_ were below therapeutic targets in all sampling periods. When compared to the non-HIV group, the ART group showed lower RIF exposure in 3T. This might be explained by EFV-driven drug-metabolic enzyme activity changes occurring during late pregnancy or by unidentified pharmacogenomic drug metabolism differences between the treatment groups. As achieving adequate TB treatment drug concentrations during pregnancy is crucial to cure TB disease and minimize adverse pregnancy and TB outcomes, the clinical relevance of low RIF exposure in PWLHIV on EFV-based ART with DS-TB disease must be determined.

## MATERIALS AND METHODS

### Study population and design

The International Maternal Pediatric Adolescent AIDS Clinical Trials Network P1026s study is a phase IV, open-label, observational, prospective study of antituberculosis and antiretroviral PK and safety in pregnant women living with and without HIV (ClinicalTrials.gov identifier NCT00042289). Recruitment sites are situated in Africa, Asia, and North and South America. PWLHIV between 20 and 38 weeks gestation receiving concurrent DS-TB treatment in either second or third trimester with EFV-based ART (the only available FDC option at the time) for at least 2 weeks were eligible to be enrolled. Completion of TB treatment could occur during pregnancy or postpartum. We excluded participants taking medications with known interactions with ART or TB treatment, participants with a clinical condition or laboratory abnormality that might require a change in the TB or ART regimen during the study period, as well as participants with multiple gestation. DS-TB treatment and ART were provided as standard of care according to WHO guidelines, and the protocol pharmacologist confirmed adequate bioequivalence of local generics prior to enrollment. The infants were enrolled *in utero* on the same day as maternal enrollment. The protocol was approved by the local institutional review boards prior to study implementation, and signed informed consent was obtained from all participants prior to participation. The participant’s local healthcare provider remained responsible for clinical management and prescription of antituberculosis treatment and ART throughout the study. Maternal safety follow-up continued until 2–8 weeks PP, and infant safety follow-up continued until 16–24 weeks of life. A previously published arm in the same study has enrolled pregnant participants without HIV on DS-TB treatment and functioned as a comparator arm with an identical study design (11) to evaluate PK differences potentially attributable to EFV exposure.

### Clinical and laboratory monitoring

Maternal demographics, medical history, concomitant medications, physical examination, and safety laboratory tests were assessed at each study visit to monitor maternal AE. Infants were also examined at 0–4 days, which included recording a Ballard score and a congenital abnormality assessment according to local standard practice, at 5–9 days, and at 16–24 weeks of life, but laboratory evaluations were only performed if clinically indicated. Where birth weight classification data were lacking, INTERGROWTH-21^st^ fetal growth standards were used to classify small for gestational age and intrauterine growth restriction (48). At each study visit, maternal and infant AEs were abstracted from clinical records from entry onward and reported per protocol guidelines, including a causality assessment for relatedness to drugs under study and monthly reviewed by the protocol team. All AEs were graded for severity according to the National Institute of Allergy and Infectious Diseases, Division of AIDS (DAIDS) Table for Grading the Severity of Adult and Pediatric Adverse Events, Version 2.0, dated November 2014 (49), and all toxicities were followed until resolution.

### Sample collection

Intensive steady-state 24 h PK profiles of RIF, INH, N-acetyl-isoniazid (ACL), EMB, and PZA were performed during the 2T (20^+0^–26^+6^ weeks gestation), 3T (30^+0^–37^+6^ weeks gestation), and 2–8 weeks PP (if still on TB treatment) in PWLHIV on EFV-based ART. Pre-dose PK blood samples were collected before directly observed daily doses of DS-TB treatment were given. All women were dosed according to WHO-recommended weight-banded dosing guidelines (10) using either fixed-dose combination or individual TB drug tablets. Further PK samples were collected at 1 h, 2 h, 4 h, 6 h, 8 h, 12 h, and 24 h post-dose. If logistically feasible at delivery, a single maternal plasma sample and an umbilical cord sample after the cord was clamped were collected. All PK samples were centrifuged, aliquoted, and stored at −70°C within 1 h of collection until analysis. A maternal dried blood spot sample for DNA extraction to determine NAT2 metabolizer genotype and detection of SLCO1B1 SNPs was collected from consenting participants.

### Assays

Plasma PK samples were analyzed at the Division of Clinical Pharmacology, University of Cape Town, South Africa, using validated high-performance liquid chromatography-tandem mass spectrometry as previously described (30) in accordance with the DAIDS Good Clinical Laboratory Practice guidelines. Respective lower limits of quantification for RIF, INH, ACL, EMB, and PZA plasma concentrations were 0.117 µg/mL, 0.105 µg/mL, 0.053 µg/mL, 0.084 µg/mL, and 0.203 µg/mL. DNA extraction was performed at Bio-Analytical Research Corporation SA laboratories, Johannesburg, South Africa, and seven NAT2 SNPs were analyzed using previously described methods (11), and the SNPedia classification (50) was used to define the NAT2 alleles. Additionally, four SLCO1B1 SNPs—rs2306283 (388A>G), rs11045819 (463C>A), rs4149056 (521T>C), and rs4149032 (38,664C>T)—were analyzed based on previous evidence of effect on RIF exposure (51, 52).

### Pharmacokinetic analyses

Similar to the previously reported pregnant comparator cohort without HIV (11), PK parameters were characterized with Stata version 16.1 (Stata Corporation, College Station, TX) using non-compartmental analyses and reviewed by the protocol pharmacologists. The steady-state AUC_0–24_ was calculated using the linear trapezoidal rule. Other PK parameters were calculated with the same approach to below quantification limit concentrations as previously reported (11) and included maximum plasma concentration (*C*_max_) and corresponding time (*T*_max_), plasma concentration at 0 h (*C*_0_), 12 h (*C*_12_), and 24 h (*C*_24_), plasma terminal half-life (*T*_½_), CL/F, and the apparent volume of distribution. The number of NAT2 acetylator gene polymorphisms identified after sequencing was used to classify maternal metabolizer status as slow, intermediate, or rapid (53). To classify metabolizer status for four participants lacking pharmacogenomic data, we made use of the 3 h ACL/INH concentration ratio as published elsewhere (54). To do so, we calculated a timepoint 3 h post-dose for ACL and INH concentrations by using the 2 h and 4 h timepoints. Detected SLCO1B1 polymorphisms were analyzed in proportion of occurrence.

### Statistical methods

Analysis of data was done using SAS version 9.4 (SAS Institute Inc., Cary, NC, USA). Demographic characteristics and clinical events were described using summary statistics, and continuous variables were reported with median and upper and lower quartiles (Q1–Q3) and categorical variables with frequencies and percentages. Descriptive statistics were summarized for PK parameters of interest during each sampling period. As pre-specified in the protocol, PK parameters during 2T vs PP and during 3T vs PP were compared at the within-participant level using the Wilcoxon signed rank test, with a two-sided *P*-value less than 0.10 considered statistically significant. Within-participant GMR and 90% CI for PK parameters in the 2T and 3T vs PP conditions were calculated for RIF and INH to assess whether there was a clinically important difference in exposure. Cross-arm between-participant GMR and 90% CI of each TB PK parameter at each corresponding sampling period was calculated by fitting univariate mixed effects models with the log-transformed TB PK parameter as the dependent variable and treatment group (ART group vs non-HIV group, with the latter as the reference group [11]) as the independent variable. Subsequently, the AUC_0–24_ and *C*_max_ were compared to pre-specified therapeutic targets described in the protocol and published literature for non-pregnant adult DS-TB patients. Acknowledging great variability in minimum efficacy AUC target estimates from previously published TB cohorts, the minimum target AUC_0–24_ for RIF, INH, and PZA selected was 35.4, 10.52, and 363 (µg·h/mL), respectively, based on analyses including bactericidal and sterilizing effect and overcoming antagonistic drug-drug interactions (8, 13, 14). No EMB AUC targets have been described in the literature. The minimum target *C*_max_ concentrations selected for RIF, INH, EMB, and PZA were 8, 3, 2, and 20 µg/mL, respectively, based on reference ranges previously published by Peloquin (12) and commonly used in several analyses (32, 55, 56). The percentages of participants with AUC_0–24_ and *C*_max_ below the minimum targets were determined during pregnancy and postpartum. AUC_0–24_ and *C*_max_ differences between NAT2 metabolizer groups were analyzed using the non-parametric Kruskal-Wallis test and proportions of SLCO1B1 SNPs between the groups with the Fisher’s exact test in Stata version 16.1 (Stata Corporation, College Station, TX) with a two-sided *P*-value less than 0.05 considered statistically significant.

## Data Availability

The data are available to all interested researchers upon request to the IMPAACT Statistical and Data Management Center’s data access committee (email address: sdac.data@fstrf.org) with the agreement of the IMPAACT Network.
